# Characterization of a Novel Capsid Assembly Modulator for the Treatment of Chronic Hepatitis B Virus Infection

**DOI:** 10.1128/aac.01348-22

**Published:** 2022-12-15

**Authors:** Dara Burdette, Anastasia Hyrina, Zhijuan Song, Rudolf K. Beran, Tara Cheung, Sarah Gilmore, Tetsuya Kobayashi, Li Li, Yang Liu, Anita Niedziela-Majka, Jonathan Medley, Upasana Mehra, Philip Morganelli, Nikolai Novikov, Congrong Niu, Danny Tam, Jennifer Tang, Jianhong Wang, Qin Yue, Simon P. Fletcher, Meghan M. Holdorf, William E. Delaney, Becket Feierbach, Scott Lazerwith

**Affiliations:** a Gilead Sciences, Inc., Foster City, California, USA

**Keywords:** capsid assembly modulator, hepatitis B virus

## Abstract

The standard of care for the treatment of chronic hepatitis B (CHB) is typically lifelong treatment with nucleos(t)ide analogs (NAs), which suppress viral replication and provide long-term clinical benefits. However, infectious virus can still be detected in patients who are virally suppressed on NA therapy, which may contribute to the failure of these agents to cure most CHB patients. Accordingly, new antiviral treatment options are being developed to enhance the suppression of hepatitis B virus (HBV) replication in combination with NAs (“antiviral intensification”). Here, we describe GS-SBA-1, a capsid assembly modulator (CAM) belonging to class CAM-E, that demonstrates potent inhibition of extracellular HBV DNA *in vitro* (EC_50_ [50% effective concentration] = 19 nM) in HBV-infected primary human hepatocytes (PHHs) as well as *in vivo* in an HBV-infected immunodeficient mouse model. GS-SBA-1 has comparable activities across HBV genotypes and nucleos(t)ide-resistant mutants in HBV-infected PHHs. In addition, GS-SBA-1 demonstrated *in vitro* additivity in combination with tenofovir alafenamide (TAF). The administration of GS-SBA-1 to PHHs at the time of infection prevents covalently closed circular DNA (cccDNA) formation and, hence, decreases HBV RNA and antigen levels (EC_50_ = 80 to 200 nM). Furthermore, GS-SBA-1 prevents the production of extracellular HBV RNA-containing viral particles *in vitro*. Collectively, these data demonstrate that GS-SBA-1 is a potent CAM that has the potential to enhance viral suppression in combination with an NA.

## INTRODUCTION

Chronic hepatitis B (CHB) is a major global health concern and one of the principal causes of chronic liver disease, cirrhosis, and hepatocellular carcinoma (HCC). The World Health Organization (WHO) estimates that in 2019, 296 million people worldwide were living with CHB, resulting in an estimated 820,000 deaths (1). Undetectable HBsAg (seroclearance) sustained for at least 6 months off treatment is the gold-standard endpoint for CHB therapy and has been associated with improvements in liver histology, including the reversal of cirrhosis, a decreased risk of HCC, and prolonged survival (2–6). The current treatments for CHB include interferon alpha (IFN-α) and nucleos(t)ide analogs (NAs). NAs provide durable on-treatment suppression of viral replication, resulting in long-term clinical benefits and a reduced risk of liver complications. As NAs do not directly target the covalently closed circular viral genome (cccDNA), treatment rarely results in the clearance of HBsAg (7–10). Furthermore, NAs are unable to completely suppress viral replication, and therefore, a low level of infectious virus persists for the majority of patients, likely contributing to the maintenance and persistence of infection (11, 12). Accordingly, new treatment options that, in combination with NAs, fully suppress hepatitis B virus (HBV) replication are potentially important components of a curative regimen.

The HBV core protein (Cp) forms viral capsids containing HBV polymerase (Pol)-bound pregenomic RNA (pgRNA), which is reverse transcribed to viral DNA. The core protein is a single polypeptide chain with a molecular weight of 20 kDa that forms dimers stabilized by extensive hydrophobic interactions among the four alpha helices located at the dimer interface. HBV core dimers further interact via their C-terminal regions, forming a trimer of dimers. This trimeric arrangement of core dimers may act as an initial scaffold upon which additional dimers spontaneously assemble into higher-order oligomers, culminating in the formation of complete HBV capsids consisting of 120 dimers (240 monomers [T4 capsid]). Capsid assembly modulators (CAMs) have been described to interact at the dimer-dimer interface, introducing small but significant perturbations in assembly. The resulting structures are malformed and unable to accommodate pgRNA replication, leading to the reduced production of infectious virions.

There are at least two classes of CAMs, known as CAM-A (e.g., heteroaryldihydropyrimidines [HAPs]) (13, 14) and CAM-E (e.g., phenyl sulfonamide-like molecules) (15–18). CAM-A produces aberrant core structures, preventing the encapsidation of pgRNA within capsids of the correct shape and size. In contrast, CAM-E induces the formation of capsids that are nicked but with a shape and size similar to those of wild-type capsids. CAMs have been proven to inhibit the secretion of both HBV DNA- and HBV RNA-containing particles (19). They can also block cccDNA establishment in cell culture systems and animal models, likely by preventing the efficient uncoating of the capsid and the delivery of HBV DNA to the nucleus, although higher CAM concentrations are required to achieve this secondary mechanism (20–22). There are a number of CAMs in clinical development, including JNJ-6379 (23, 24), ABI-H0731 (25, 26), RO7049389 (27), and GLS4-JHS (28). More potent molecules are also being developed to achieve exposures that would effectively block both new virus production and the establishment of cccDNA.

Here, we describe GS-SBA-1, a CAM-E that shows potent, pangenotypic inhibition of extracellular HBV DNA and RNA both *in vitro* and *in vivo*. Additionally, *in vitro* profiling demonstrates potent inhibition of cccDNA formation and activity against nucleos(t)ide-resistant mutants. As GS-SBA-1 acts by a mechanism orthogonal to that of NAs, the combination of these compounds has the potential to effectively block viral spread by stopping viral replication and preventing the reinfection of new cells.

## RESULTS

### GS-SBA-1 alters capsid assembly.

**FIG 1:**
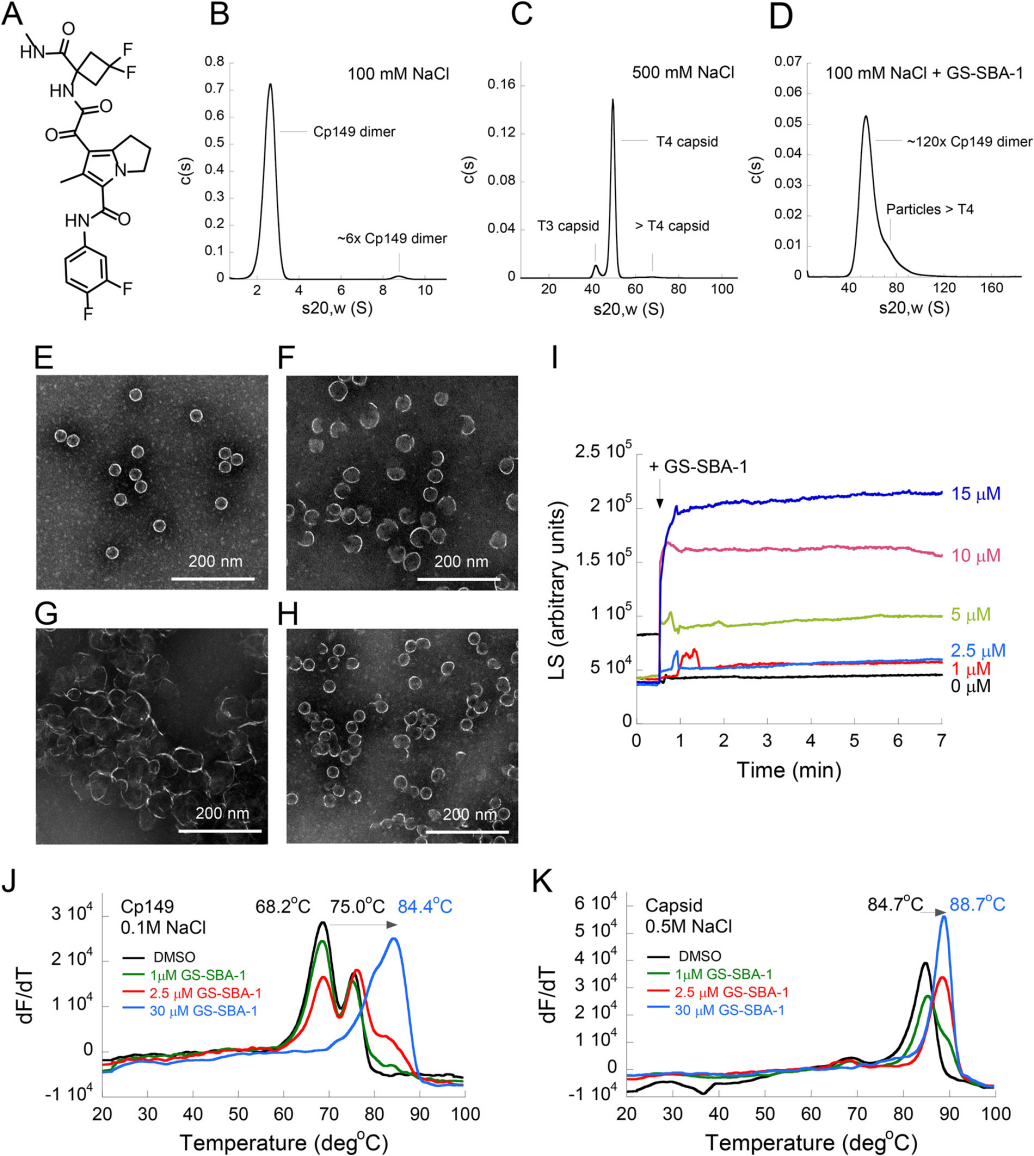
GS-SBA-1 alters *in vitro* capsid assembly. (A) Chemical structure of GS-SBA-1. (B to D) Sedimentation coefficient distributions for Cp149 dimers (B), *in vitro*-assembled T4 and T3 capsids (C), and capsid particles (D), where assembly was induced by the binding of GS-SBA-1 to Cp149 dimers. AUC in sedimentation velocity mode was performed at 20°C with a solution containing 7.5 μM Cp149 dimers in a buffer containing 50 mM HEPES (pH 7.5), 2 mM DTT, and 0.5% DMSO supplemented with 100 mM NaCl (B), 500 mM NaCl (C), or 100 mM NaCl (D) and a stoichiometric amount of GS-SBA-1 (15 μM). (E to H) Representative EM micrographs of Cp149 dimers assembled in the presence of 0.5% DMSO (E), a stoichiometric amount of GS-SBA-1 (F), a 1.3-fold molar excess of HAP12 (G), or a 1.3-fold molar excess of NVR-3-778 (H) at room temperature in a buffer containing 50 mM HEPES (pH 7.5), 2.5 mM DTT, and 500 mM NaCl. (I) Representative LS curves of the assembly of Cp149 dimers into capsid particles were collected in the presence of 1% DMSO or GS-SBA-1 (final concentrations are indicated in the graph). GS-SBA-1 or DMSO was added to a solution of 3 μM Cp149 dimers in a buffer containing 50 mM HEPES (pH 7.5), 300 mM NaCl, and 2 mM DTT at 37°C. The time of compound addition is marked by an arrow. (J and K) Representative thermograms obtained for 3 μM Cp149 dimers (J) or *in vitro*-assembled capsids (K) in the absence (1% DMSO [black]) or presence (1 μM [green], 2.5 μM [red], and 30 μM [blue]) of GS-SBA-1. The DSF assay was performed using a buffer containing 50 mM HEPES (pH 7.5), 2.5 mM DTT, 1% DMSO, and either 100 mM NaCl (J) or 500 mM NaCl (K). Arrows indicate the direction of peak shifts. A first derivative transformation of a fluorescence (F) as a function of a temperature (T) was plotted (dF/dT).

Biochemical and cellular biological studies were performed to understand the mechanism of action of GS-SBA-1 (Fig. 1A) on capsid assembly modulation. Analytical ultracentrifugation (AUC), negative-stain electron microscopy (EM), 90° light scattering (LS), and differential scanning fluorimetry (DSF) were used to monitor the effect of GS-SBA-1 on the assembly of the core protein Cp149. Cp149 is commonly used in biochemical studies due to its high homogeneity and the dependence on only protein-protein interactions for capsid assembly (29). Full-length Cp has 183 amino acid residues and can be functionally and structurally divided into two main domains: an amino-terminal capsid assembly domain encompassing amino acids 1 to 149 (Cp149) and a C-terminal RNA binding domain containing stretches of basic residues (30). The N-terminal domain is necessary and sufficient to drive the self-assembly of Cp *in vitro* at micromolar protein concentrations and in the presence of high salt concentrations at neutral pH (31).

AUC experiments demonstrated that in the presence of 100 mM NaCl, Cp149 was mostly dimeric (98.5% of the protein; sedimentation coefficient [*s*_20,_*_w_*] = 2.66 S), with a small amount of low-molecular-weight oligomers (likely an assembly of six dimers; 1.5% of the protein) (Fig. 1B). Consistent with previous reports, Cp149 dimers self-assembled into high-molecular-weight capsid particles at NaCl concentrations of >500 mM (Fig. 1C) (32). Two types of well-defined particles were detected by AUC at 500 mM NaCl: a dominant form (89.9% of the protein), which had an *s*_20,_*_w_* value of 49.5 S, and a minor form (7.2% of the protein), which had a lower *s*_20,_*_w_* value of 41.5 S. Based on *s*_20,_*_w_* values and in agreement with published data, these two species were assigned as capsids with T4 and T3 icosahedral symmetries, respectively. The observed ratio of T3 to T4 capsids is consistent with previous reports and reflects the proportion found in core particles *in vivo* (31, 33). The addition of a stoichiometric amount of the capsid assembly modulator GS-SBA-1 to Cp149 dimers in the presence of 100 mM NaCl induced the quantitative assembly of core dimers into a mixture of high-molecular-weight particles with a broad distribution of particle sizes (Fig. 1D). Some of these particles had sizes comparable to or slightly larger than that of the T4 capsid (84.5% of the protein; *s*_20,_*_w_* = 54.4 S), whereas the rest (15.1% of the protein) were larger multimers (*s*_20,_*_w_* = 70.9 S) (Fig. 1D). Hence, GS-SBA-1 binds to the HBV core protein and induces its assembly into high-order structures under conditions that are normally not permissive for assembly (100 mM NaCl).

The assembled particles were analyzed further by EM. In agreement with published reports, Cp149 capsids obtained at 500 mM NaCl in the absence of GS-SBA-1 were uniformly spherical, with a mean diameter of 29 ± 1 nm (Fig. 1E) (30, 33, 34). The particles obtained at 500 mM NaCl in the presence of GS-SBA-1 had highly variable shapes and dimensions, with a mean diameter of 33 ± 5 nm (Fig. 1F). Many of them were nonspherical and larger than regular T4 capsids; some displayed nicks or breaks in a spheroid-like shape or had spiral-like shapes. Particles for which assembly was induced by the addition of GS-SBA-1 at 100 mM NaCl had a similar appearance and comparable average dimensions (mean diameter, 35 ± 6 nm [data not shown]) to those obtained at 500 mM NaCl in the presence of GS-SBA-1. This suggests that GS-SBA-1 directs the assembly of a core protein into particles with an aberrant morphology different from that of Cp149 particles, which assemble in the absence of the compound. The Cp149 particles assembled in the presence of HAP12, a CAM-A (35), and NVR-3-778, a CAM-E (36), also exhibited altered sizes, being substantially larger (55 ± 14 nm) (Fig. 1G) and slightly smaller (26 ± 3 nm) (Fig. 1H) than a regular T4 capsid, respectively. These data suggest that GS-SBA-1 behaves more like CAM-E in that that they both drive the formation of spheroid-like particles with nicks and breaks and sizes that are on average larger or smaller than that of a capsid assembled in the absence of the compound; however, the morphology of particles can be somewhat affected by the conditions under which assembly is performed.

The kinetics of the assembly of Cp149 dimers into particles was profiled by light scattering (LS) No increase above the background was observed in the absence of GS-SBA-1 under conditions that are not permissive for the self-assembly of Cp149 dimers (low protein concentrations and low NaCl concentrations) (Fig. 1I) or upon the addition of GS-SBA-1 to the buffer in the absence of Cp149 dimers (data not shown). The addition of GS-SBA-1 to a solution of Cp149 dimers, however, induced a rapid and compound-concentration-dependent increase in LS (Fig. 1I), suggesting that GS-SBA-1 increases the extent and rate of incorporation of Cp149 dimers into high-molecular-weight capsid particles.

Finally, the *in vitro* binding of GS-SBA-1 to free Cp149 dimers and preformed capsids was assessed by DSF, which measures the temperature of the thermal unfolding transition of a protein (*T_m_*) based on the change in the fluorescence intensity of an environmentally sensitive fluorophore (SYPRO orange). To study the effect of GS-SBA-1 binding on core protein assembly, DSF experiments were performed on Cp149 dimers at 100 mM NaCl. In the absence of GS-SBA-1, Cp149 dimers display two melting transitions, with *T_m_* values equal to 68.2°C ± 0.2°C (major) and 75.0°C ± 0.2°C (minor) (Fig. 1J). The first transition likely represents the thermal denaturation of a Cp149 dimer, whereas the second transition may represent the unfolding of a Cp149 dimer with an intramolecular disulfide bond, as this transition becomes more pronounced upon prolonged storage in which Cp149 slowly oxidizes. The addition of GS-SBA-1 demonstrated compound-concentration-dependent changes in the melting profile: a decrease in the amplitude of the first transition with the concomitant appearance of a new peak with a high *T_m_* and a shift of the second transition to higher temperatures. At concentrations of GS-SBA-1 equal to or higher than those of Cp149 monomers, which are necessary for capsid particle formation, only one asymmetric peak is visible, with a *T_m_* value of 84.4°C ± 0.2°C, representing compound-bound particles with variable sizes.

We further profiled the binding of GS-SBA-1 to *in vitro*-assembled capsids by DSF utilizing a buffer containing 500 mM NaCl, for which assembled capsid particles are stable (*T_m_* of 84.7°C ± 0°C) (Fig. 1K). The addition of GS-SBA-1 resulted in a shift to a higher *T_m_* (Δ*T_m_* = 4.0°C), which suggests that GS-SBA-1 binds to and stabilizes the assembled Cp149 capsid particles (Fig. 1K). Altogether, biochemical mechanism-of-action (MOA) studies indicate that GS-SBA-1 has an activity similar to that of the CAM-E, leading to the accelerated formation of a mixture of morphologically altered capsids.

### GS-SBA-1 has potent antiviral activity in primary human hepatocytes.

**FIG 2:**
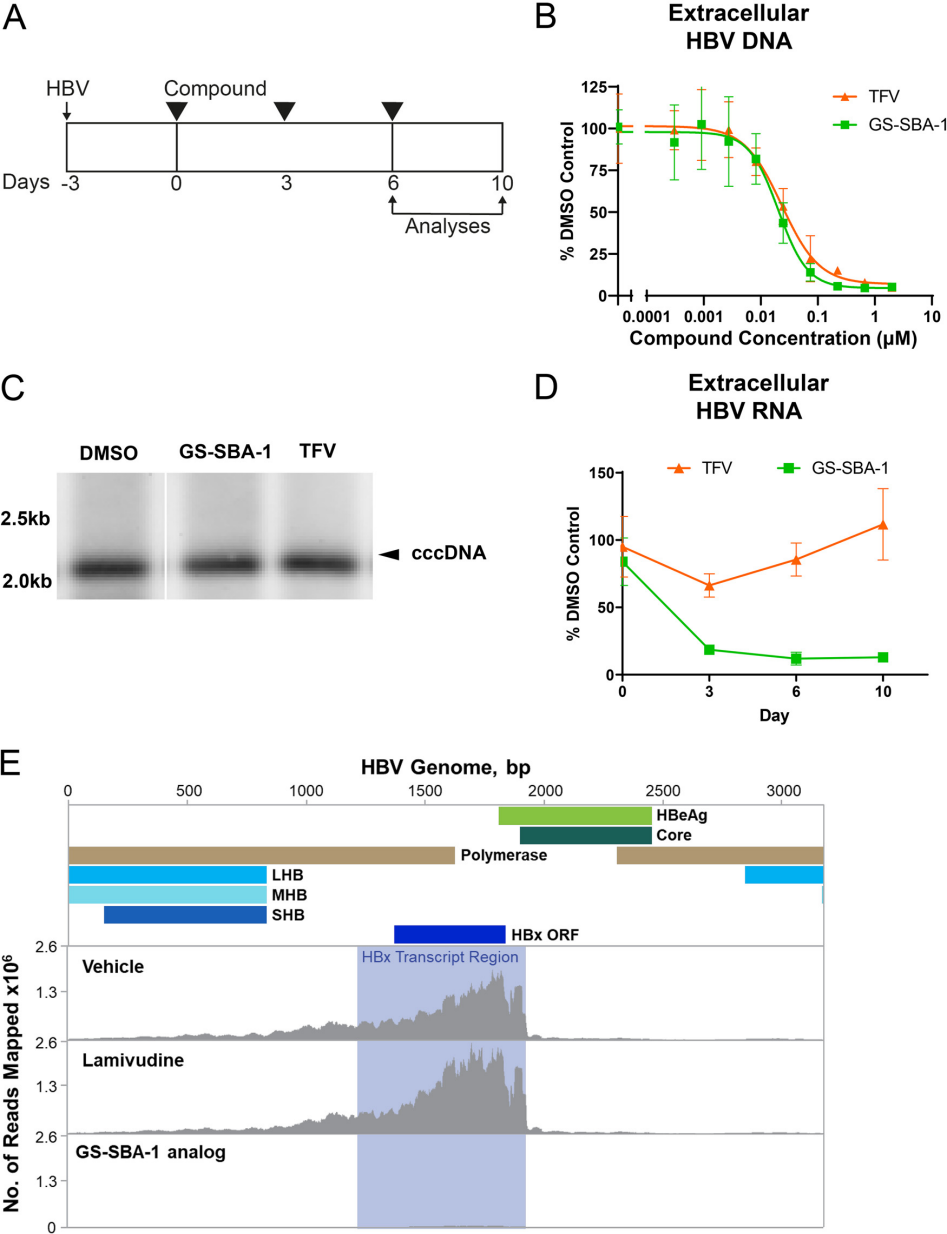
Antiviral activity of GS-SBA-1 in primary human hepatocytes. (A) Schematic of the antiviral assay in PHHs. (B) Representative dose-response curves of extracellular HBV DNA for TFV and GS-SBA-1. HBV DNA is shown as a percentage relative to the DMSO-only control. Data are shown as means ± standard deviations (SD). (C) HBV-infected PHH cells were treated with the indicated compounds at 50× EC_50_ for 6 days as depicted in panel A. Total cellular DNA was isolated, and Southern blotting was performed. An image representative of results from two independent experiments is shown. (D) Time course of GS-SBA-1 and TFV treatment on extracellular HBV RNA. Extracellular HBV RNA is shown as a percentage relative to the DMSO-only control. Data are representative of results from two independent experiments with at least two biological replicates and are shown as means ± SD. (E) RNAseq of polyethylene glycol (PEG)-precipitated HBV particles from HepAD38 cell supernatants treated with a GS-SBA-1 analog (GS-4110) and lamivudine at 50× EC_50_ or the DMSO control for 21 days after tetracycline removal. Reads are plotted by alignment to the linear HBV genome. ORF, open reading frame; LHB, large HBV surface protein; MHB, middle HBV surface protein; SHB, small HBV surface protein.

The antiviral activity of GS-SBA-1 was evaluated in primary human hepatocytes (PHHs) isolated from four different donors who were infected with HBV genotype D (GTD), according to the schematic in Fig. 2A. The antiviral activity of GS-SBA-1 was compared to that of tenofovir (TFV), a nucleotide analog that inhibits HBV replication. Both TFV and GS-SBA-1 potently inhibited HBV DNA production across donors (EC_50_ [50% effective concentration] = 0.030 ± 0.0077 μM and EC_50_ = 0.019 ± 0.0071 μM, respectively) in the absence of cytotoxicity at the concentrations tested in this assay (Fig. 2B; see also Table S1 in the supplemental material). No effect on intracellular HBV RNA or extracellular HBV antigen levels was observed (EC_50_ of >2 μM) (data not shown). GS-SBA-1 was similarly efficacious in HBV-infected HepG2-NTCP cells (HBV DNA EC_50_ = 0.0093 ± 0.0035 μM) (Table S1).

The effects of GS-SBA-1 and TFV on cccDNA and extracellular HBV RNA levels in GTD HBV-infected PHHs were also evaluated at 50-fold over the mean HBV DNA EC_50_ (50× EC_50_). Neither GS-SBA-1 nor TFV had a significant effect on cccDNA levels in HBV-infected PHHs as evaluated by Southern blotting (Fig. 1C). Similar results were observed for HBV-infected HepG2-NTCP cells (data not shown). In contrast, extracellular HBV RNA was potently inhibited by GS-SBA-1, while minimal inhibition was observed for TFV on day 3 only (Fig. 1D). To further characterize the extracellular HBV RNA, we profiled HBV particles purified from HepAD38 cells (37) treated with a GS-SBA-1 analog (GS-4110) and the nucleoside analog lamivudine (LAM) at 50× EC_50_ for 21 days by transcriptome sequencing (RNAseq). Consistent with previously reported data, we detected viral reads that mapped predominantly within the HBx transcript region in vehicle-treated samples as well as some longer reads (Fig. 1E) (38). HepAD38 cells treated with lamivudine contained levels of HBV RNA reads similar to those in the control samples; however, HBV preparations derived from GS-SBA-1-treated HepAD38 cells contained substantially fewer HBV RNA reads (Fig. 1E).

To determine if the HBV genotype impacts the antiviral potency of GS-SBA-1, PHHs were infected with GTA, GTB, GTC, GTD, or GTE HBV isolated from HBV-infected patients as shown in Fig. 2A. Consistent with the high core protein amino acid conservation (39), GS-SBA-1 had similar antiviral activities across all tested genotypes (EC_50_ values ranged from 0.0080 μM to 0.017 μM) (Table 1). Similarly, the antiviral activities of the nucleotide analog tenofovir alafenamide (TAF) were comparable across all tested genotypes (Table 1).

**TABLE 1:** Pangenotypic antiviral activity of GS-SBA-1 in PHHs treated in a therapeutic format

Genotype of patient isolate^*a*^	Patient ID	Avg extracellular HBV DNA EC_50_ (μM)^*c*^	
		GS-SBA-1	TAF
GTA	91P	0.014	0.00031
GTB	Lab derived^*b*^	0.0081	ND
GTC	27	0.0091	0.00026
GTC	05	0.0080	0.00013
GTD	65P	0.012	0.00026
GTE	30P	0.017	0.00029

a Calculated by 4-parameter logistic curve fitting of data from a 6-day treatment of HBV-infected PHHs. Averages from two independent experiments are shown.

b The genotype B virus is a cell culture-derived virus concentrated from cells expressing a genotype B consensus sequence.

c ND, not determined.

### GS-SBA-1 retains potency against nucleos(t)ide-resistant HBV.

The emergence of resistance mutations in HBV polymerase has been observed clinically with the NAs lamivudine and adefovir (ADV). The mutations V173L, L180M, and M204I/V are conferred by lamivudine, while adefovir resistance is conferred by the mutations A181V and N236T (40, 41). To determine whether nucleos(t)ide resistance mutations confer cross-resistance to GS-SBA-1, stable cell lines expressing wild-type (WT) HBV (GTA), ADV-resistant HBV (AVNT-3-39 [GTA], encoding the A181V and N236T mutations), or LAM-resistant HBV (VLLMMV24 [GTA], encoding the V173L, L180M, and M204V mutations) were treated with GS-SBA-1 as well as ADV, LAM, and TAF as controls for 5 days (Table 2). GS-SBA-1 demonstrated similar activity against the nucleos(t)ide-resistant HBV strains (EC_50_ values within 2-fold of those of the wild type) (Table 2). Consistent with previously reported data, ADV- and LAM-resistant HBV strains showed reduced levels of susceptibility to ADV, LAM, and TAF (40, 41). Collectively, these results indicate a lack of cross-resistance between NAs and GS-SBA-1.

**TABLE 2:** Antiviral activity of GS-SBA-1 against nucleos(t)ide-resistant HBV strains

Treatment	Antiviral activity against extracellular HBV DNA				
	Wild-type mean EC_50_ (μM)^*a*^	ADV-resistant strains		LAM-resistant strains	
		Mean EC_50_ (μM)^*a*^	Fold resistance^*b*^	Mean EC_50_ (μM)^*a*^	Fold resistance^*b*^
GS-SBA-1	0.0090	0.010	1.1	0.014	1.6
ADV	16	300	19	33	2.1
LAM	0.17	43	250	120	705
TAF	0.034	0.60	18	0.10	2.9

a Calculated by 4-parameter logistic curve fitting of data from a 5-day treatment of stable cell lines expressing wild-type HBV or mutants resistant to the nucleoside analog adefovir (ADV) or lamivudine (LAM). Averages from two independent experiments are shown.

b Calculated as mutant EC_50_/wild-type EC_50_.

### GS-SBA-1 blocks cccDNA establishment.

**FIG 3:**
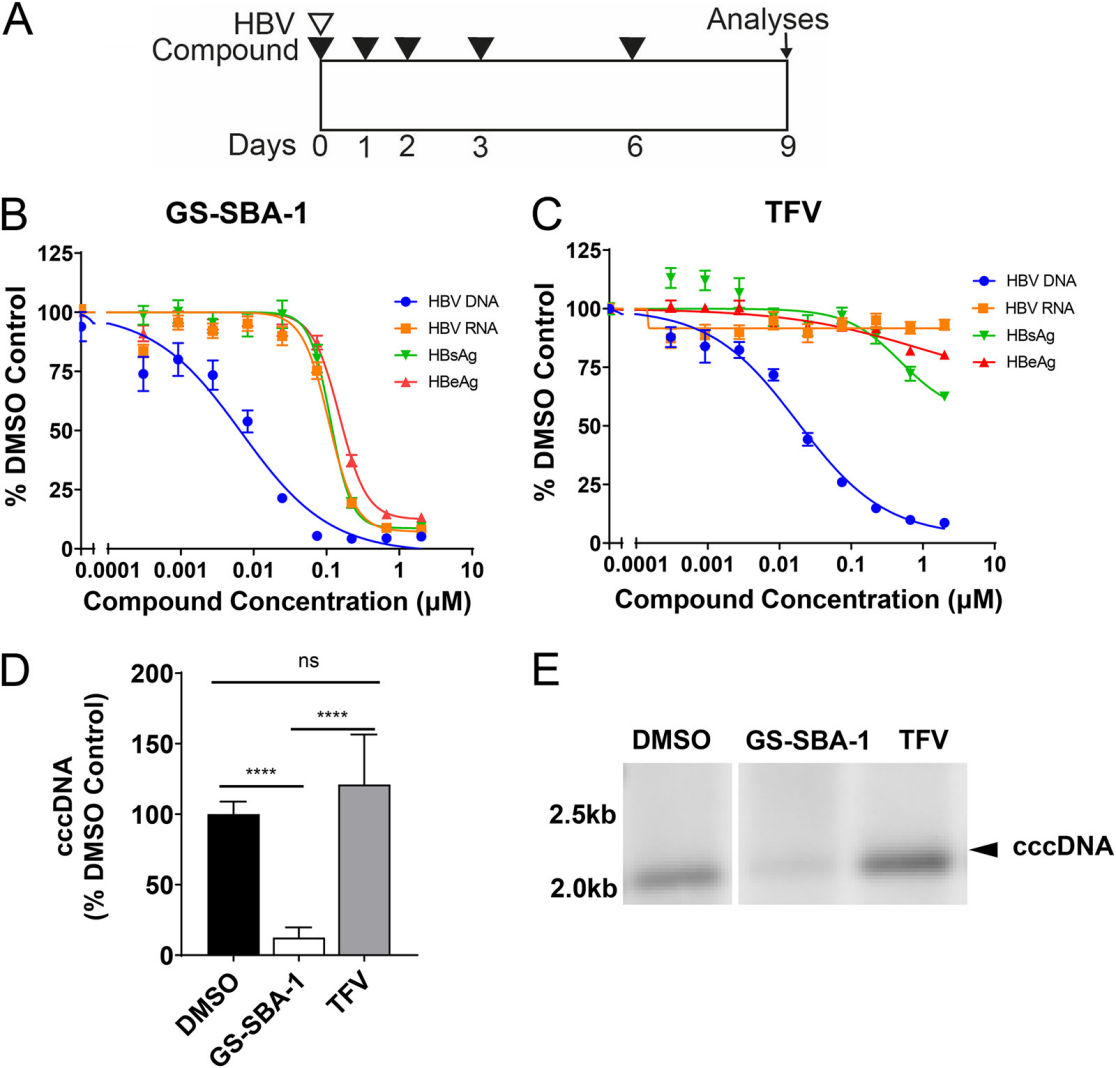
Antiviral activity of GS-SBA-1 against the early stage of the HBV life cycle in PHHs. (A) Schematic of the antiviral assay to measure the effect of compounds on the early stage of the HBV life cycle in PHHs. (B and C) Dose-response curves for extracellular HBV DNA, intracellular HBV RNA, HBeAg, and HBsAg in PHHs from 4 donors treated with GS-SBA-1 (B) and TFV (C). HBV DNA, RNA, and antigens are shown as percentages relative to the DMSO-only control. Symbols represent the means, and error bars represent the SD. (D and E) PHH cells were treated with the indicated compounds at 50× EC_50_ as depicted in panel A. (D) qPCR analysis of T5-digested total cellular nucleic acids amplified with primer-probe sets targeting the direct repeat DR1-DR2 region. Data are averages with SD from two independent experiments with at least two biological replicates. Statistical analysis was performed using the Mann-Whitney nonparametric two-tailed *t* test. ****, *P* < 0.0001; ns, not significant (*P* > 0.05). (E) Southern blotting of total cellular nucleic acids. An image representative of results from two independent experiments is shown.

Based on biochemical evidence showing the stabilization of HBV capsids by GS-SBA-1, we hypothesized that GS-SBA-1 could prevent the efficient uncoating of the capsid and the delivery of relaxed circular DNA (rcDNA) to the nucleus, thereby blocking cccDNA establishment. To examine this, PHHs were treated with GS-SBA-1 or TFV at the time of HBV infection, as outlined in Fig. 3A. GS-SBA-1 and TFV inhibited the production of extracellular HBV DNA when added together with the viral inoculum (0 h), with a potency similar to that when added at 72 h postinfection (Fig. 2B, Fig. 3B and C, and Table S2). GS-SBA-1, but not TFV, also inhibited intracellular HBV RNA (EC_50_ = 0.12 μM) and the production of the HBV antigens HBeAg (EC_50_ = 0.17 μM) and HBsAg (EC_50_ = 0.14 μM) (Fig. 3B and C and Table S2) across multiple PHH donors when added at 0 h postinfection.

To determine if the inhibition of HBV RNA and antigen production was the result of a block in cccDNA establishment, PHHs were treated with 50× HBV DNA EC_50_ of GS-SBA-1 or TFV as shown in Fig. 3A, and cccDNA was quantified by quantitative PCR (qPCR) and Southern blotting. In this assay format, GS-SBA-1, but not TFV, resulted in a reduction of cccDNA levels as measured by qPCR (*P* < 0.0001 versus the dimethyl sulfoxide [DMSO] control) and Southern blotting (Fig. 3D and E). These results suggest that early treatment with GS-SBA-1 blocks the establishment of cccDNA in the nucleus, resulting in reductions in HBV RNA transcription and antigen production.

### GS-SBA-1 demonstrates antiviral efficacy in a liver-chimeric mouse model of HBV infection.

**FIG 4:**
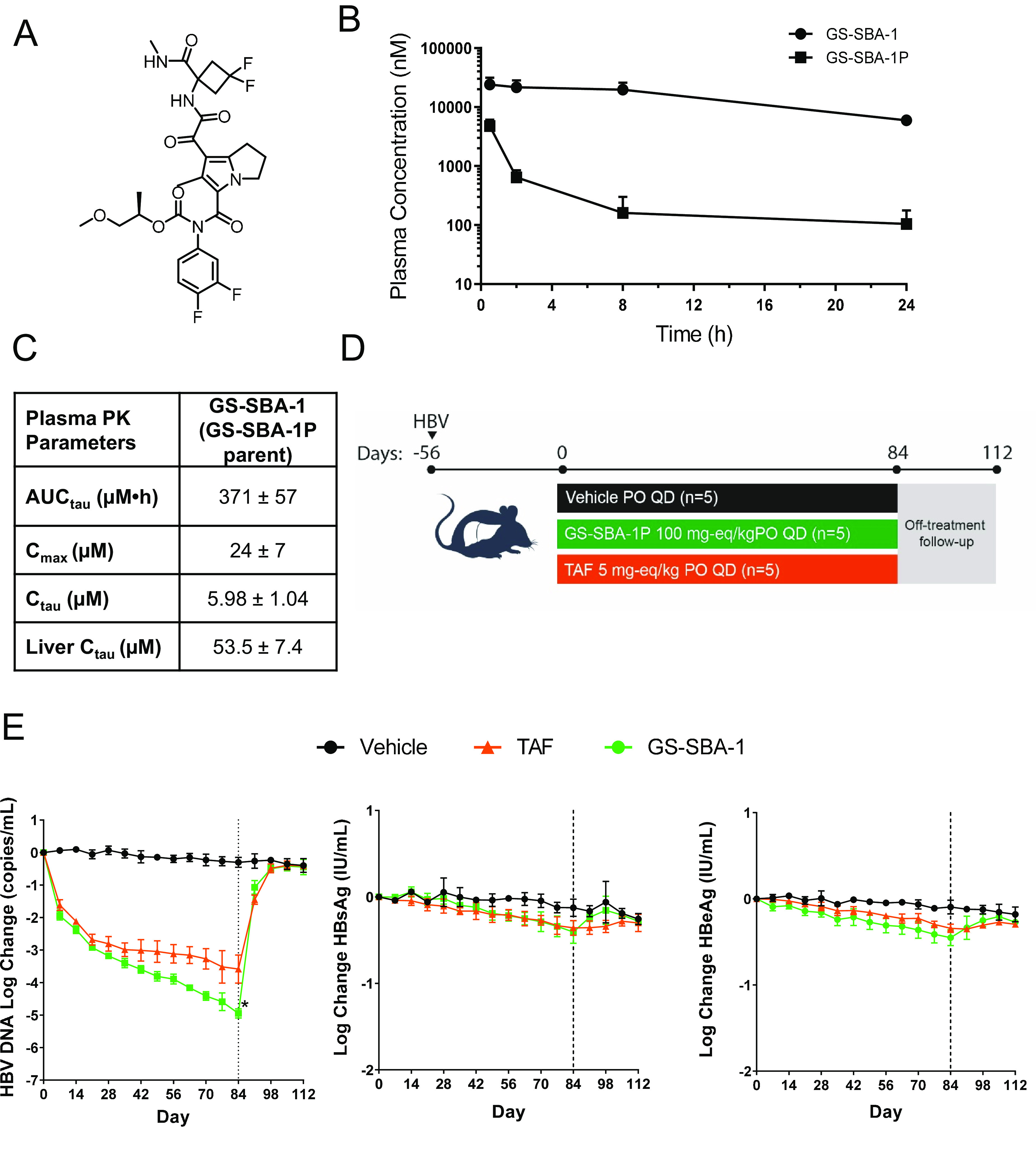
Antiviral efficacy of GS-SBA-1 in HBV-infected uPA/SCID mice. (A) Chemical structure of GS-SBA-1P (GS-SBA-1 prodrug). (B and C) Uninfected uPA/SCID mice were dosed with 100 mg eq/kg GS-SBA-1P once daily for 14 days. (B) Concentrations of GS-SBA-1P and GS-SBA-1 in mouse plasma. Plasma samples were collected 0.5 h, 2 h, 8 h, and 24 h following the last dose of GS-SBA-1P. (C) Pharmacokinetic (PK) parameters (AUC_τ_, *C*_max_, *C*_τ_, and liver *C*_τ_) (means with minimum and maximum limits). (D) Schematic of the GS-SBA-1 efficacy study in HBV-infected uPA/SCID mice. Fifteen uPA/SCID mice were infected with genotype C HBV. After 8 weeks of infection, mice were dosed with 100 mg eq/kg GS-SBA-1P or 5 mg eq/kg TAF (*n* = 5 per group) for 84 days. Mice were monitored for an additional 28 days. PO, orally; QD, once a day. (E) Longitudinal analysis of serum HBV DNA, HBsAg, and HBeAg levels during treatment and after treatment follow-up. All data are presented as means ± SD. *, below the lower limit of quantitation.

The uPA/SCID mouse model was used to profile the *in vivo* antiviral activity of GS-SBA-1. Plasma samples were collected 0.5, 2, 8, and 24 h following the last dose of the prodrug GS-SBA-1P. The concentrations of the prodrug GS-SBA-1P and its parent GS-SBA-1 in the plasma were determined by liquid chromatography-tandem mass spectrometry (LC-MS/MS). The area under the concentration-time curve from time 0 to 24 h (AUC_τ_), the maximum concentration of the drug in serum (*C*_max_), and the *C*_τ_ of GS-SBA-1 were 371 μM · h, 24 μM, and 5.98 μM, respectively (Fig. 4B and C). The GS-SBA-1P plasma exposure was less than 3% of that of its parent GS-SBA-1, with the conversion of the prodrug to GS-SBA-1 being driven primarily by CYP3A4 in the liver (data not shown). The oral administration of GS-SBA-1P resulted in high steady-state trough concentrations of GS-SBA-1 in the plasma, efficiently covering severalfold above the protein-adjusted EC_95_ (paEC_95_) of GS-SBA-1 (paEC_95_ = 564 nM). The protein-adjusted EC_95_ was calculated based on parameters from the *in vitro* HBV-infected PHH studies (EC_50_ = 0.015 μM; Hill slope = 1.49) together with the calculated plasma protein shift (plasma shift = 5.1) as determined by equilibrium dialysis through a semipermeable membrane against 100% human serum.

To evaluate the antiviral efficacy of GS-SBA-1, uPA/SCID mice (*n* = 15) were infected with genotype C HBV for 8 weeks to allow the viral kinetics to reach steady state prior to treatment with 100 mg eq/kg of body weight of GS-SBA-1P or 5 mg eq/kg of TAF (Fig. 4D). Mice were dosed orally once daily for 84 days and monitored for an additional 28 days. Serum HBV DNA and HBV antigen levels were measured weekly throughout the study. By day 84 (end of treatment), the mean reductions of serum HBV DNA were −3.6 log_10_ units with TAF and −4.95 log_10_ units with GS-SBA-1P (Fig. 4E). Serum HBV DNA levels rebounded rapidly following treatment cessation for both compounds. Minor reductions in both HBsAg and HBeAg were also observed with both treatments. TAF and GS-SBA-1P reduced serum HBsAg by −0.36 log_10_ and −0.41 log_10_ units, respectively; similar reductions were observed for HBeAg (Fig. 4E). Treatment with GS-SBA-1P was well tolerated; mice did not experience any changes in body weight or blood albumin and experienced only minimal changes in serum alanine transaminase (Fig. S1A to C).

### GS-SBA-1 demonstrates antiviral additivity in combination with TAF.

**FIG 5:**
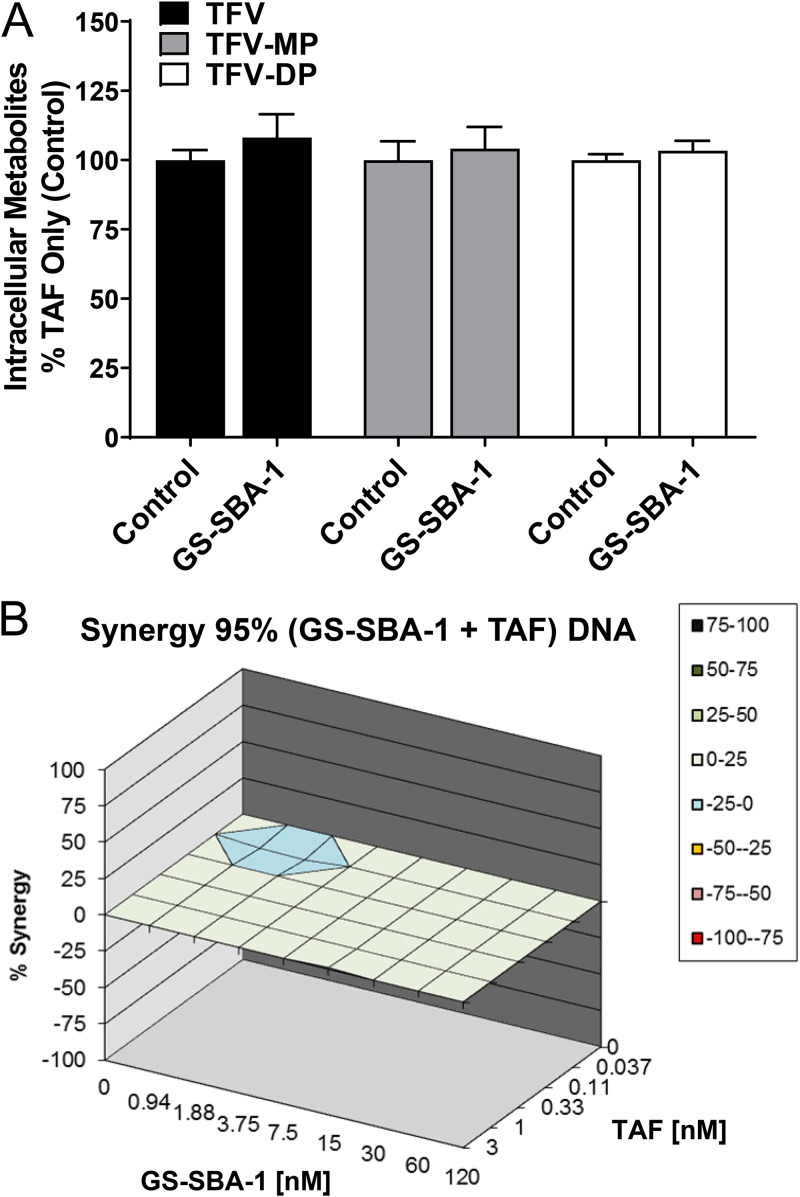
Analyses of the synergy between GS-SBA-1 and TAF. (A) Intracellular metabolite levels for 0.5 μM TAF alone (control) or in combination with 1.5 μM GS-SBA-1 after 24 h. Levels were determined in three different PHH donors by a standard curve and are expressed as a percentage relative to the TAF-only control. Error bars represent means ± SD. (B) PHHs were infected with HBV genotype D at day 0. After the establishment of infection, PHHs were treated with serially diluted compounds on days 3 to 9 for a total of 6 days. The EC_50_ value of each compound was selected as the midpoint for the concentration range tested. A representative synergy plot was calculated using MacSynergy II software. The synergy plot (95% confidence) of extracellular HBV DNA for combination treatment with GS-SBA-1 and TAF is shown. Data are representative of results from three independent experiments.

As capsid modulators are likely to be used in combination with an NA in the clinic, we profiled the intracellular metabolism of TAF in the presence and absence of GS-SBA-1. Fresh PHHs were incubated with 0.5 μM TAF alone or in combination with 1.5 μM GS-SBA-1 for 24 h, and the intracellular concentrations of TFV and its phosphorylated metabolites (TFV monophosphate [TFV-MP] and TFV diphosphate [TFV-DP]) were determined by LC-MS/MS. GS-SBA-1 had no effect on the accumulation of TFV or the phosphorylated metabolite TFV-MP or TFV-DP (Fig. 5A).

To assess the compatibility of GS-SBA-1 and TAF in antiviral assays, HBV-infected PHHs were treated with each drug alone or in combination for 6 days (days 3 to 9 postinfection), and extracellular HBV DNA was measured. MacSynergy II software was used to analyze antiviral drug interactions based on the Bliss independence model by quantifying statistically significant differences between the theoretical and observed HBV DNA inhibition values (42). Plotting these differences in three dimensions results in a surface where elevations in the z-plane represent antiviral synergy and depressions represent antiviral antagonism between compounds. The calculated volumes of surface deviations are expressed in μM^2^%. Based on three independent experiments in triplicate, TAF in combination with GS-SBA-1 had an average synergy score of 15 ± 25 and an average antagonism score of −18 ± 29, indicating that the two compounds have additive antiviral activity when combined (Fig. 5B).

## DISCUSSION

As the majority of CHB patients on long-term NA therapy likely still have ongoing active viral replication, orthogonal agents that can be combined with NAs to fully suppress HBV replication and prevent viral spread are being developed (12). Small molecules targeting HBV capsid assembly have demonstrated the efficient inhibition of pgRNA encapsidation, leading to a reduction of extracellular HBV DNA in cell culture systems, *in vivo* mouse models, and CHB patients. Therefore, targeting capsid assembly is an attractive antiviral approach for further reducing the production of new viral particles in combination with NAs. In addition, targeting the capsid provides the further benefit of preventing the production of extracellular HBV RNA-containing particles and potentially blocking infection of naive hepatocytes.

In this study, we describe the preclinical characterization of GS-SBA-1, a potent and selective capsid assembly modulator. GS-SBA-1 potently inhibited the production of HBV virions with similar antiviral activities across the four PHH donors tested and demonstrated additivity with TAF *in vitro*. In addition, GS-SBA-1 inhibits cccDNA establishment as well as the downstream viral products (viral RNA, HBeAg, and HBsAg) when added at the time of infection. In comparison to the antiviral activity against viral DNA production, a 16- to 19-fold-higher compound concentration is required to affect the early stage of the HBV life cycle and inhibit cccDNA formation. The differential compound concentrations required for the primary and secondary antiviral mechanisms are similar to those reported previously for the CAMs JNJ-632 and BAY41-4109 and can be explained by the CAM MOA (21). For antiviral activity against viral DNA production, CAMs bind to a hydrophobic pocket formed by two core protein dimers, accelerating the kinetics of capsid assembly and preventing Pol-pgRNA encapsidation. For inhibiting the early stage of the HBV life cycle, CAMs are likely binding to the preformed capsids, stabilizing and preventing their disassembly and, consequently, the release of the rcDNA template used for cccDNA establishment. Higher concentrations are required for the latter mechanism as CAMs need to bind to multiple sites to stabilize a functional capsid structure.

In addition to inhibiting the production of HBV virions, we show that GS-SBA-1, but not TAF, can also prevent the production of extracellular HBV RNA-containing particles. We and others have demonstrated that circulating HBV RNA particles, which can contain various HBV RNAs, including HBx transcripts, are present in cell culture-derived virus preparations and the serum of CHB patients (38, 43, 44). Circulating HBV RNAs have been linked with an increased risk of HCC (45, 46). It has been suggested that HBV RNAs may directly promote HCC development through interactions with host oncogenes and/or increased intrahepatic inflammation and fibrosis (47, 48). HBV DNA integration, which is considered to play a role in HCC development, may also lead to the production of circulating HBV RNAs (43). Additionally, HBV RNA transcripts potentially could directly express HBx protein upon infection in a cccDNA-independent manner to support cccDNA transcription by antagonizing host restriction factors such as the SMC5/6 complex (38, 44). The reduction of extracellular HBx transcripts by CAM, but not by a polymerase inhibitor, is a differentiating feature of these compound classes and awaits further investigation of its potential role in HBV infection.

GS-SBA-1P, a prodrug of GS-SBA-1, also significantly reduced serum HBV DNA by 4.95 log_10_ units, compared to 3.6 log_10_ units with TAF alone, in a humanized liver-chimeric mouse model of HBV, consistent with *in vitro* profiling. Despite achieving exposures >1,500-fold higher than the mouse plasma-adjusted EC_50_ for HBV DNA production (150-fold higher than the intracellular HBV RNA EC_50_ when dosed prophylactically), GS-SBA-1P showed no significant declines in HBsAg after 84 days of dosing. It is possible that the duration of the study was not long enough to observe a sufficient turnover of HBV-infected hepatocytes as there is no immune component to drive the clearance of infected hepatocytes in the immunocompromised uPA/SCID mouse model.

The core protein sequence is highly conserved across consensus sequences of all known HBV genotypes (genotypes A to H). In both assays of HBV-infected PHHs and transfection assays in HepG2 cells, GS-SBA-1 showed similar activities across all genotypes, including the known ADV- and LAM-resistant HBV strains, within 2.5-fold of the reference values. The patient polymorphisms T33N and Y118F were previously described to be resistant to GS-SBA-1 (EC_50_s >240-fold and 3-fold higher than those of the wild type, respectively) and other capsid assembly modulators (49, 50). Sequence analyses suggested that these mutations are prevalent in <0.3% of the patient population (49). However, monotherapy with a CAM may result in resistant variants, including T33N, requiring combination treatment with an NA (51).

Collectively, these data demonstrate that GS-SBA-1 acts by a mechanism orthogonal to that of NAs and has additive antiviral activity when combined with TAF *in vitro*. The antiviral additive effect will need to be further confirmed in an animal model, as others have shown that not all CAM molecules result in a pronounced reduction of the antiviral response in combination with an NA compared to monotherapy in the uPA/SCID mouse model (52). Recent studies have demonstrated that HBV integration rates are closely linked to HBV DNA levels (53–55); therefore, it is anticipated that enhanced antiviral suppression would be beneficial to reduce potential oncogenic HBV integration events. GS-SBA-1 also demonstrates a potent reduction of HBV RNA-containing particles, potentially inhibiting the transcription of newly formed cccDNA. Taken together, these data support the further development of GS-SBA-1 in combination with TAF.

## MATERIALS AND METHODS

### Compounds.

Tenofovir alafenamide fumarate (TAF), tenofovir (TFV), TFV-DP, GS-SBA-1, GS-SBA-1P, lamivudine, GS-4110, HAP12 (35), and NVR-3-778 (36) were synthesized by the Medicinal Chemistry Department at Gilead Sciences, Inc.

### Cell lines.

HepAD38 cells were grown in Dulbecco’s modified Eagle’s medium (DMEM)–F-12 medium (catalog number 10565018; Thermo Fisher Scientific) supplemented with 10% fetal bovine serum (catalog number 10082147; Thermo Fisher Scientific), 1% penicillin-streptomycin-glutamine (catalog number 10378016; Thermo Fisher Scientific), 1% HEPES (catalog number 15630080; Thermo Fisher Scientific), and 1% nonessential amino acids (catalog number 11140050; Thermo Fisher Scientific) (37). Human hepatoblastoma (HepG2) cells stably expressing the HBV receptor Na^+^ taurocholate cotransporting polypeptide (NTCP) (HepG2-NTCP cells) were generated internally and cultured as previously described (56). The HepG2 cell line was obtained from the American Type Culture Collection (ATCC) (Manassas, VA) and was maintained in DMEM–F-12 medium (Invitrogen, Carlsbad, CA) supplemented with 10% heat-inactivated fetal bovine serum (Invitrogen, Carlsbad, CA) and 1× penicillin-streptomycin-glutamine (Invitrogen, Carlsbad, CA). HepG2 cells stably expressing wild-type HBV, LAM-resistant HBV (VLLMMV24 [41]), and ADV-resistant HBV (AVNT-3-39 [40]) were maintained in DMEM–F-12 growth medium (catalog number 10565-018; Thermo Fisher Scientific) containing 10% fetal bovine serum (catalog number SH30071.03, lot number AVK85745; Thermo Fisher Scientific), 1× penicillin-streptomycin (catalog number 15140-122; Thermo Fisher Scientific), 1× Geneticin (G418) (catalog number 10131-027; Thermo Fisher Scientific), 1× HEPES (catalog number 15630-080; Thermo Fisher Scientific), and 1× minimal essential medium (MEM) nonessential amino acids (catalog number 11140-050; Thermo Fisher Scientific).

### Expression of Cp149.

Escherichia coli BL21(DE3) cells transformed with an expression construct encoding an N-terminal capsid assembly domain (amino acids 1 to 149) of core protein (Cp149) from HBV genotype D strain AD38 were grown at 37°C with shaking at 150 rpm overnight. This culture was used to inoculate 6 L of Luria broth (LB) containing ampicillin and chloramphenicol (0.1 and 0.033 mg/mL, respectively) at an initial *A*_600_ of 0.05. The culture was grown at 37°C at 150 rpm until it reached an *A*_600_ of 0.6. Protein expression was then induced by the addition of isopropyl 1-thio-β-d-galactopyranoside to a final concentration of 0.5 mM. The culture was shaken at 170 rpm for an additional 4 h at 37°C. Cells were pelleted by centrifugation, and the pellet was stored at −80°C.

### Purification of Cp149.

Twenty grams of the cell pellet was lysed in 200 mL of lysis buffer (50 mM Tris [pH 7.5], 150 mM sucrose) by passage through a microfluidizer. The homogenate was centrifuged at 30,000 × *g* for 60 min at 4°C. Ammonium sulfate was added to the supernatant to a final concentration of 0.91 M. The sample was stirred slowly at 4°C for 1 h and then centrifuged for 1 h at 4°C at 30,000 × *g*. The supernatant was discarded, and the ammonium sulfate pellet was solubilized in 100 mL phosphate-buffered saline (PBS). The solubilized pellet was centrifuged at 30,000 × *g*, and the supernatant containing Cp149 (11 mg/mL) was stored at −80°C. Cp149 was diluted to 1 mg/mL with 0.1 M sodium bicarbonate buffer (pH 9.6) containing 3.5 M urea and incubated for 3 h at room temperature. Cp149 was loaded onto a Superdex S200 gel filtration column equilibrated in a buffer containing 0.1 M sodium bicarbonate (pH 9.6) and 2 mM dithiothreitol (DTT). Dimer-containing fractions of Cp149 in a buffer containing 0.1 M sodium bicarbonate (pH 9.6) and 2 mM DTT were pooled and concentrated to 1 mg/mL.

A detailed description of the methods used can be found in the supplemental material.

### Ethical statement.

All animal protocols were performed in accordance with the Guide for the Care and Use of Laboratory Animals and approved by the Animal Welfare Committee of Phoenix Bio Co., Ltd. All mice were housed individually and maintained in accordance with the Animal Ethics Committee of PhoenixBio (resolution #2214).
