# Supplementary material for: Characterization of a Novel Capsid Assembly Modulator for the Treatment of Chronic Hepatitis B Virus Infection
Source: Antimicrob Agents Chemother. 2022 Dec 15;67(1):e01348-22. doi: 10.1128/aac.01348-22 (PMC9872672; doi:10.1128/aac.01348-22)
Supplement: Supplemental file 1 — Supplemental material. Download aac.01348-22-s0001.pdf, PDF file, 0.2 MB [file aac.01348-22-s0001.pdf]

Supplemental Materials and Methods

Analytical Ultracentrifugation

Analytical ultracentrifugation (AUC) sedimentation velocity experiments (AUC) of Cp149 dimer in the absence and presence of GS-SBA-1 were performed at 20°C in a ProteomeLab XL-A analytical ultracentrifuge (Beckman Coulter. Fullerton, CA). 7.5 μM of Cp149 dimer alone or with 15 μM of GS-SBA-1 was incubated for 1 hour at room temperature in buffer containing 50 mM Hepes, pH 7.5, 100 mM NaCl, 2 mM DTT, and 0.5% DMSO before subjecting to the analysis. Alternatively, 7.5 μM of Cp149 dimer was incubated in the presence of high salt (500 mM NaCl) in buffer containing 50 mM Hepes, pH 7.5, 2 mM DTT, and 0.5% DMSO. Samples of 400 μL were loaded into a dual sector charcoal-filled Epon centerpiece and centrifuged at 20,000 rpm in an An50-Ti rotor. In experiments performed with GS-SBA-1, compound at the same final concentration was placed in the reference channel of the centerpiece. Sedimentation was monitored by absorbance at a wavelength of 280 nm. Sedimentation velocity absorbance scans were analyzed with the program SEDFIT (NIH, Bethesda, MD), which generates a continuous c(s) distribution for the sedimenting species (1). Calculated apparent sedimentation coefficient values (s) were converted to the standard conditions of water at 20°C ($s_{20,w}$) using SEDFIT. The program SEDENTERP (2) was used to estimate the partial specific volume of the proteins as well as the density and viscosity of the buffer solution.

Transmission Electron Microscopy

Cp149 dimers were assembled into particles in the absence or in the presence of capsid assembly modulators by incubation in buffer containing 50 mM Hepes, pH 7.5, 2 mM DTT, 500 mM NaCl, and 0.5% DMSO at room temperature. Cp149 dimers were incubated at a concentration of 10 μM or 7.5 μM, and compounds were tested at either an equimolar concentration (20 μM of GS-SBA-1) or 1.3-fold molar excess (20 μM of HAP12 or NVR-3-778) with respect to Cp149 dimer. Transmission electron microscopy (EM) was performed on negative stained samples within 20 hours of particle assembly. Samples were deposited onto

parlodion filmed carbon-coated grids and embedded in 2% potassium phosphotungstate at pH 6.5 using the drop method. Negatively stained specimens were examined at 80 kV in a JEOL JEM-1230 electron microscope (JEOL USA Inc., Peabody, MA) and photographed with a Gatan Ultrascan USC1000 digital camera (Gatan Inc., Warrendale, PA). Dimensions of particles were quantified by analysis of EM micrographs with the program ImageJ (NIH, Bethesda, MD). On average 240 to 700 objects over 7 to 8 individual micrographs taken for each sample were measured.

Ninety-degree Light Scattering Experiments

The kinetics of assembly of Cp149 dimers into capsid particles were measured by light scattering (LS). LS experiments were performed using a Photon Technology International fluorimeter (Edison, NJ) with excitation and emission wavelengths set at 400 nm with a 3 nm band-pass and detection of scattered light at 90°. At this wavelength, the reaction components showed no significant absorption of the light. Cp149 dimers at 3 μM concentration were preincubated for 10 minutes at 37°C in buffer containing 50 mM Hepes, pH 7.5, 300 mM NaCl, 2 mM DTT to allow for temperature equilibration. Assembly was initiated by addition of GS-SBA-1 at 1, 2.5, 5, 10 and 15 μM concentrations or 1% DMSO (for the reaction performed in the absence of compound). All concentrations are final after mixing. Scattering was initially measured for 30 seconds for a sample containing only Cp149 dimer, followed by compound addition, mixing, and further LS measurement for an additional 370 seconds with stirring of the reaction mixture. LS data were also collected in the absence of Cp149 dimers to check for possibility of background light scattering due to any potential aggregation and/or precipitation of GS-SBA-1 under the experimental conditions. No scattering was observed for GS-SBA-1 at each concentration used in the assay. All measurements were made at 37°C using a black masked microcuvette with a 0.3 cm path length (Hellma, Müllheim, Germany). The scattered light was attenuated by a neutral density filter (Starna, Atascadero, CA). Raw fluorescence intensities of scattered light (arbitrary units) were plotted as a function of time for assembly reactions containing either 1% DMSO (no compound) or variable concentrations of GS-SBA-1 without further analysis.

Differential Scanning Fluorimetry

Capsid particles were assembled by addition of 500 mM NaCl to a solution of 40 μM of Cp149 dimers in buffer containing 25 mM Hepes, pH 7.5, and 2.5 mM DTT, followed by incubation for 2 hours at room temperature. All concentrations are final after mixing. Differential scanning fluorimetry (DSF) measures the midpoint of the thermal unfolding transition of a protein (melting temperature, T_m) based on the change in fluorescence intensity of an environmentally sensitive fluorophore such as SYPRO Orange. Specific binding of a compound to the protein results in an increase of protein thermal stability. At a given protein and compound concentration, the increase in T_m (ΔT_m) is proportional to the compound equilibrium association constant, allowing the use of DSF to confirm compound binding (3). DSF experiments were performed at 3 μM of Cp149 dimer or assembled capsid diluted to 3 μM (concentration in Cp149 dimer) and at 1, 2.5 and 30 μM of GS-SBA-1 in 1% DMSO (or 1% DMSO alone in the absence of compound). Protein and compound were pre-incubated for 1 hour at room temperature in buffer containing 50 mM Hepes, pH 7.5, 2.5 mM DTT, and 100 mM NaCl or 500 mM NaCl for studies with Cp149 dimer or assembled capsid, respectively. SYPRO Orange was added at 1:1000 (v/v) dilution. All concentrations were final after mixing. Thermal denaturation was performed by increasing the temperature from 20°C to 99.5°C at 1°C/minute on a ViiA7 Real-Time PCR instrument (Life Technologies, Carlsbad, CA). Fluorescence intensity data were collected at intervals of 0.07°C. The assay was performed in the absence and presence of compounds. Fluorescence intensity data were analyzed with Protein Thermal Shift Software (Life Technologies, Carlsbad, CA) using a first derivative approach to calculate T_m. The change in protein melting temperature (ΔT_m) was calculated by subtracting the T_m value obtained for protein in the presence of 1% DMSO (no compound) from the T_m value obtained in the presence of a compound and 1% DMSO.

HepAD38 Virion Production

Supernatant containing virions from HepAD38 cells cultured in the absence of tetracycline was collected every 3 to 4 days and virions were precipitated with PEGit (Systems Biosciences, Palo Alto, CA; LV825A-

1) overnight at 4°C. Following precipitation, supernatant was spun at 3000 rpm at 4°C for 15 minutes and the pellet containing the virions was resuspended in William's E medium (Thermo Fisher Scientific, A1217601) containing 25% fetal bovine serum (Thermo Fisher Scientific, 10082147). The viral titer was determined by quantitative PCR (qPCR).

Genotype A, B, C, D, and E HBV Patient-derived Viruses

Plasma from an HBV genotype A (GTA) infected patient (016GT33791P), genotype D (GTD) infected patient (036LS27465P), and genotype E (GTE) infected patient (024KY12630P) were purchased from Proteogenex (Culver City, CA). Plasma from an HBV genotype C infected patient (GTC-27; 89461-10693-45599-20131227) or sera from an HBV genotype B (GTB) infected patient (7737-10693-36784020130520) and HBV GTC infected patient (GTC-05; 56662-27867-39729-20130905) were purchased from BioCollections, Worldwide, Inc. (Miami, FL).

PHH Culture Conditions and HBV Infection

Cryopreserved primary human hepatocytes (PHH) isolated from multiple donors were purchased from Thermo Fisher Scientific (Waltham, MA; HMCPTS, Donors Hu8234, Hu1748) or BioreclamationIVT (Hicksville, NY; Donor YJX, IYP). After thawing, cells were recovered by centrifugation at 100 x *g* (Beckman Coulter, Allegra X-14R) through cryopreserved hepatocyte recovery medium (Thermo Fisher Scientific; CM7500) and plated in collagen coated 96-well plates (Thermo Fisher Scientific; CM1096) at a density of 55,000 to 75,000 live cells per well. Cells were plated in William's E medium (Thermo Fisher Scientific; A1217601) supplemented with 3.6% hepatocyte thawing and plating supplement (Thermo Fisher Scientific, A15563), 5% fetal bovine serum (Thermo Fisher Scientific; 16000-036), 1 μM dexamethasone (Thermo Fisher Scientific, A15563), and 0.2% Torpedo antibiotic mix (BioreclamationIVT; Z990008). Approximately 12 – 14 hours after plating, plating medium was removed and cells were switched into maintenance medium: William's E medium (Thermo Fisher Scientific; A1217601) supplemented with 4% hepatocyte maintenance supplement (Thermo Fisher Scientific; AI15564), 2% fetal bovine serum (Thermo

Fisher Scientific; 16000-036), 0.1 μM dexamethasone (Thermo Fisher Scientific; AI15564), 1.5% DMSO (Sigma-Aldrich, St. Louis, MO; D8418), and 0.2% Torpedo antibiotic mix (BioreclamationIVT; Z990008). Approximately 24 hours after plating, PHH were infected with HepAD38-derived HBV virions (4) (genotype D virus) at 500 viral genome equivalents per cell in maintenance medium supplemented with 4% PEG 8000 (Promega, Madison, WI; V3011). The infection was allowed to proceed for 20 – 24 hours before removing remaining extracellular virions by washing with maintenance medium 3 times.

HepG2-NTCP Culture Conditions and Bulk Infection

HepG2-NTCP cells were seeded in T175 flasks 3 days prior to infection in basic growth medium (DMEM medium (Thermo Fisher Scientific, 11965-092), 200mM, Thermo Fisher Scientific, 25030-081)) containing 10% fetal bovine serum (Thermo Fisher Scientific, SH30071.03, lot AVK85745) and 0.25 mg/mL G418 (Thermo Fisher Scientific, 10131035). On Day 0, cells were infected with 4000 viral genome equivalents per cell HepAD38-derived HBV virions (4) (genotype D virus) generated as described above in basic growth media containing 2% FBS and 2.5% DMSO (Sigma, D8418) supplemented with 4% PEG 8000 (Promega, Madison, WI; V3011). The infection was allowed to proceed for 20 – 24 hours before removing remaining extracellular virions by washing with OptiMEM (Thermo Fisher Scientific, 31985070) 3 times. On Day 4, cells were removed from the flask using 0.1% trypsin (Thermo Fisher Scientific, 15400054) and trypsinization was halted by adding basic growth medium. Cells were centrifuged at 1200 rpm for 5 minutes (Beckman Coulter, Allegra X-14R). Cells were subjected to 2 additional wash steps with OptiMEM and resuspended after the last wash in antiviral assay medium (basic growth media containing 2% FBS and 1% DMSO) at 0.2 x 10^6 cells/mL. Cells were plated in collagen coated 96-well plates (Thermo Fisher Scientific; CM1096) at a density of 30,000 cells per well and treated with compounds.

Compound Treatment

Three or four days following infection of PHH or HepG2-NTCP cells, respectively, with AD38 virus (Day 0), maintenance medium was replenished and cells (in triplicate wells) dosed with compound supplied in

100% DMSO using the HP Digital Dispenser D300 (Hewlett Packard, Palo Alto, CA). HBV-infected PHH and HepG2-NTCP cells were dosed with compounds using serial dilutions ranging from 2 μM to 0.31 nM. DMSO was used as the vehicle control. Vehicle and compound containing wells were normalized such that the final concentration of DMSO was 1.7% (v/v). Medium containing compound was removed and PHH or HepG2-NTCP cells were re-dosed with new media containing the appropriate compounds on Day 3 following the initial dose for a total of 2 doses. On Day 6 post-initiation of dosing for both PHH and HepG2-NTCP culture systems, supernatant and cells collected to assess antiviral activity and cytotoxicity.

Compound Treatment Assays at the Time of HBV Infection in PHH

HepAD38-derived HBV virions were dosed with compounds using serial dilutions ranging from 2 μM to 0.31 nM and incubated for 1 hour at room temperature. Approximately 24 hours after plating in collagen coated 96-well plates (Thermo Fisher Scientific; CM1096) at a density of 55,000 to 75,000 live PHH cells per well, medium was removed and HepAD38-derived HBV virions in the presence of compounds were transferred from v-shaped 96-well plates to PHH-containing 96-well plates. The infection was allowed to proceed for 20 – 24 hours in triplicate wells for each compound concentration before removing remaining extracellular virions by washing with William's E medium 3 times. Maintenance medium was replenished, and cells were dosed with compound supplied in 100% DMSO using the HP Digital Dispenser D300. Maintenance media with tested compounds were replenished for total of 3 times on Days 1, 3, and 6 after the infection.

Measurement of HBeAg and HBsAg Concentrations by Immunoassay

Extracellular hepatitis B virus S antigen (HBsAg) and hepatitis B virus E antigen (HBeAg) were detected in culture media by an electrochemiluminescence assay (MSD) as previously reported (5).

Quantification of Extracellular DNA by qPCR

Viral DNA from PHH supernatants was purified using the Qiagen DNeasy 96 kit (69582) following the manufacturer's recommended protocol. Quantification of vDNA by qPCR (quantitative polymerase chain reaction) amplification of the HBx region of the genome was performed by combining 5 µL of DNA, 900 nM of HBx forward primer, 900 nM of reverse primer (Supplemental Table 3), 0.2 µM TaqMan probe, and 1x TaqMan Fast Advanced Master Mix (Thermo Fisher Scientific; 4444557) for a total reaction volume of 20 µL in 96-well PCR plates (Thermo Fisher Scientific; 4346906). qPCR was carried out on a real-time PCR system (Thermo Fisher Scientific; QuantStudio 7 Flex) using the following conditions: 95°C for 20 seconds, followed by 40 cycles of 95°C for 1 second and 60°C for 20 seconds. A plasmid containing the HBV full genome was used for the standard curve.

Quantification of Cytotoxicity

Cytotoxicity was measured by alamarBlue® (Thermo Fisher Scientific, DAL1100). Following removal of all culture supernatant, cells were replenished with maintenance (PHH) or antiviral assay (HepG2-NTCP) medium containing 10% (v/v) alamarBlue® dye. Cells were incubated at 37ºC for 3 hours. Fluorescence was measured using a fluorescence excitation wavelength of 540 nm and fluorescence emission was read at 585 nm on a spectrophotometer (Molecular Devices, SpectraMax M5) and analyzed with SoftMax Pro 6.3 software (Molecular Devices).

Quantification of Intracellular Viral RNA

Intracellular HBV viral RNA (vRNA) was isolated from PHH using the RNeasy 96 kit (Qiagen, 74182) following the manufacturer's recommended protocol. Quantification of vRNA by qRT-PCR (quantitative reverse transcription polymerase chain reaction) amplification of the HBVX region of the genome was performed by combining 5 µL of RNA to 900 nM of HBVX forward primer, 900 nM of HBVX reverse primer (Supplement Table 4), 0.2 µM TaqMan probe (Supplemental Table 4), and 1x beta-actin (ACTB) endogenous transcripts (Thermo Fisher Scientific; 4310881E) and 1x TaqMan Fast Virus 1-Step Master Mix (Thermo Fisher Scientific; 4444434) for a total reaction volume of 20 µL in 96-well PCR plates

(Thermo Fisher Scientific; 4346906). qRT-PCR was carried out on a real-time PCR system (Thermo Fisher Scientific; QuantStudio 7 Flex) using the following conditions: 50°C for 5 minutes, then 95°C for 20 seconds, followed by 40 cycles of 95°C for 3 seconds and 60°C for 30 seconds. ACTB mRNA expression was used to normalize target gene expression. Levels of HBV mRNA for all donors were calculated as fold change relative to no drug treated sample using the $2^{-\Delta\Delta Ct}$ method (6).

Data Analysis

Antiviral activity or cytotoxicity of each test compound was determined from vRNA, vDNA, HBeAg, HBsAg, and alamarBlue® data by comparing compound-treated PHH to DMSO-treated PHH to generate a percent of DMSO control value (% DMSO control). The % DMSO control was calculated by the following equation: % DMSO Control = 100 x (XC / XD) where XC is the signal from the compound-treated PHH and XD is the signal from the DMSO-treated PHH. The % DMSO control for vRNA, vDNA, HBeAg, HBsAg, and alamarBlue® was plotted versus the log of each compound concentration in GraphPad Prism (version 6; GraphPad Software, La Jolla, CA) to generate dose-response curves. EC_{50} values were defined as the test compound concentration that caused a 50% decrease in vRNA, vDNA, HBeAg, or HBsAg. CC_{50} values were defined as the test compound concentration that caused a 50% decrease in alamarBlue®. The top of the dose-response curves was constrained to 100 and fitted using the nonlinear regression equation "log(agonist) versus response – Variable slope (4 parameters)" in GraphPad Prism to determine EC_{50} or CC_{50} values. Error bars represent standard deviation. Data are representative of 2 independent experiments in PHH and 4 independent experiments in HepG2-NTCP cells. The geometric means were calculated from EC_{50} or CC_{50} values from independent experiments within each PHH donor and reported as a factor (x or ÷) of the geometric deviation.

HBV Cellular DNA Extraction and Purification

Extraction of HBV cellular DNA was carried out using precipitation procedure which was described in detail by manufacturer's instruction (EPICENTRE; MC85200) with minor modifications. Briefly, cells in

24- well plate were washed twice with 1× PBS and lysed in 0.5 mL of Tissue and Cell Lysis Solution. After 10 minutes incubation at room temperature, the lysate was transferred into a 1 mL tube, followed by the addition of 0.25 mL of MPC Protein Precipitation Reagent. After 10 minutes on ice, the lysate was clarified by centrifugation (Eppendorf; Centrifuge 5430R) at ≥10,000 × g for 10 minutes at 4°C. The supernatant was transferred to a clean microcentrifuge tube and DNA extracted with 0.75 mL of isopropanol by centrifugation at ≥10,000 × g for 10 minutes at 4°C. HBV cellular DNA pellet was rinsed twice with 1 mL of 70% ethanol and dissolved in 100 μL of TE buffer. Extracted HBV cellular DNA was purified using Zymo-Spin™ IIC Column which was described in detail by manufacturer's instruction (ZYMO Research; D4034). Briefly, 100 μL of HBV cccDNA was mixed with 200 μL of DNA Binding Buffer, transferred into the Zymo-Spin™ IIC column in a collection tube, and centrifugation (Eppendorf; Centrifuge 5415D) at ≥10,000 × g for 30 seconds. HBV cccDNA bound to the membrane was washed twice with 300 μL of DNA Wash Buffer. After washing, the column was transferred to a clean 1.5 mL microcentrifuge tube. HBV cccDNA was eluted with 30 μL of DNA Elution Buffer.

HBV cccDNA Quantitation by qPCR

HBV cellular DNA was treated with T5 exonuclease (New England Biolabs; M0363S) according to manufacturer's instructions prior to quantification by qPCR. HBV cccDNA was quantified by real-time PCR using 1× TaqMan Fast Advanced Master Mix (Thermo Fisher Scientific; 4444557) on real-time PCR system (Thermo Fisher Scientific; QuantStudio 7 Flex). PCR was carried out in the following conditions: initial denaturation for 20 seconds at 95°C, followed by 40 cycles at 95°C denaturation for 1 second and annealing/elongation at 60°C for 20 seconds. cccDNA specific primers which targeted DR1-DR2 gap region (Supplemental Table 4) were used throughout this study. HBV cccDNA copy number was quantified relative to a linearized plasmid DNA standard.

Southern Blotting Analysis

Purified HBV cellular DNA samples were resolved in a 1.2% agarose gel (Roche; 11388983001) in 1× TAE buffer (TEKnova; T1260). After electrophoresis, the DNA was depurinated, denatured (Biosciences; R016), and neutralized (Biosciences; R018) exactly as described by Cai et al. (7) and transferred onto Nytran SuPerCharge membrane (GE Healthcare, 10416216) using the TurboBlotter system (GE Healthcare; 10416300). The membrane was hybridized with an HBV-DNA probe by branched DNA signal amplification (bDNA) method. All reagents for the bDNA signal amplification method were from Affymetrix. Briefly, transfer membranes were pre-hybridized with lysis mixture (cat# QG0504) and blocking reagent (cat#QS0505) at 55°C for 30 minutes. After, HBV DNA target probe, QG (cat# QS1051), was added and allowed to hybridize overnight at 55°C. The next day, pre-Amplifier (2.0 PreAmp cat# QG15905), amplifier (2.0 Amp cat# QG15098) and label probe containing alkaline phosphatase (2.0 Label Probe cat#QG1324) were added sequentially. Pre-amplifier and amplifier mixtures were incubated at 55°C for 1 hour while the label probe was incubated at 50°C for 1 hour. Between each incubation step the membrane was washed 3 times with QuantiGene Wash Buffer (cat# QG0509). Following bDNA hybridization procedures, HBV DNA was detected using CDP-Star (GE Healthcare; NIF1229) according to manufacturer's instructions. Images were acquired with ImageQuant LAS 4000 (GE Healthcare) with exposure for a few second to minutes. Density of cccDNA bands was determined using ImageQuant TL software (version 7, GE Healthcare).

RNA-Seq analysis

RNA-Seq was conducted by Q2 Expression Analysis (Durham, NC) as described previously (5). Briefly, extracellular RNA was isolated using TRizol (Thermo Fisher Scientific) from HepAD38 cells supernatants treated with DMSO control or compounds at 50X EC50. cDNA libraries were constructed using a TruSeq Stranded mRNA Library Prep Kit (Illumina, San Diego, CA). Pair-end sequencing was conducted using Illumina HiSeq2000 with read length of 50 nucleotides. On average, approximately 30 million reads were generated per sample. Sequencing reads were aligned to the human and HBV genomes by STAR method

(8). RNASeq data were deposited in SRA with BioProject accession PRJNA906647 (https://www.ncbi.nlm.nih.gov/bioproject/PRJNA906647).

Wild-Type, Nucleos(t)ide Resistant Stable Cell Line Assay

Human hepatoblastoma cells (HepG2) stably expressing wild-type HBV, LAM-resistance HBV (VLLMMV24; (9)) and ADV-resistance (AVNT-3-39; (10)) were plated on Day 0 in collagen coated 96-well plates (Thermo Fisher Scientific; CM1096) at a density of 25,000 cells per well in DMEM-F12 growth medium. On Day 0, cells were dosed in triplicate with compound supplied in 100% DMSO using the HP Digital Dispenser D300 (Hewlett Packard, Palo Alto, CA). HBV wild-type, LAM-resistant and ADV-resistant cells were dosed with compounds using serial dilutions ranging from 50 µM to 0.128 nM. DMSO was used as the vehicle control. Vehicle and compound containing wells were normalized such that the final concentration of DMSO was 0.5% (v/v). After 5 days of incubation with compound, extracellular viral DNA (vDNA) was quantified to assess compound EC50.

Pharmacokinetic Studies

To evaluate pharmacokinetics of GS-SBA-1 at steady state, uninfected uPA-SCID mice were treated orally with 5 mg-eq/kg TAF or 100 mg-eg/kg of GS-SBA-1P, a prodrug of GS-SBA-1 once daily for 14 days. Plasma samples were collected at 0.5 h, 2 h, 8 h, 24 h post dose following last dose of GS-SBA-1P. The concentrations of prodrug GS-SBA-1P and its parent GS-SBA-1 in the plasma were determined by liquid chromatography–tandem mass spectrometry. Pharmacokinetic parameters, including area under the plasma concentration–time curve from time 0 to 24 hour (AUC_{tau}), maximal concentration (C_{max}) and concentration at 24 hours following last dose (C_{tau}) were determined by non-compartmental analysis using Phoenix WinNonlin 6.4 (Pharsight Corporation, Princeton, NJ).

uPA-SCID Mouse Model

Male uPA/SCID mice between 12-18 weeks of age with humanized liver (cDNA-uPA$^{wild/+}$/SCID [cDNA-uPA$^{wild/+}$: B6;129SvEv-Plau, SCID:C.B-17/Icr-scid /scid Jcl) were produced as previously described by PhoenixBio, Co. Ltd. (Japan) (11). Briefly, frozen human hepatocytes (donor BD195, Corning Incorporated, Tewksbury, MA, USA) were thawed and transplanted into 2- to 4-week-old uPA/SCID mice by splenic injection. Mice were selected for studies if their liver reconstitution levels had an estimated replacement index greater than 70% based on the blood concentration of human albumin (>8.5mg/mL) one week prior to study initiation. General health observations including weight were monitored weekly. All animal protocols were performed in accordance with the Guide for the Care and Use of Laboratory Animals and approved by the Animal Welfare Committee of Phoenix Bio Co., Ltd. All mice were housed individually and maintained in accordance with the Animal Ethics Committee of PhoenixBio (resolution #2214).

HBV-infected mice were randomized into three different treatment groups based on body weight, blood h-Alb, and serum HBV DNA concentrations. All mice had blood h-Alb levels above 12 mg/mL and serum HBV DNA levels above 8.8×10^8 copies/mL. Mice received an oral dose of 100 mg-eq/kg of GS-SBA-1P, 5 mg-eq/kg of TAF or dosing vehicle (vehicle control) once daily for 84 days. GS-SBA-1P was dosed in 1% (w/w) HPMC and 0.3% (w/w) polysorbate 80 in water, while TAF was dosed in 10 mM phosphate buffer, pH 6.5. Mice were followed for an additional 28 days following cessation of treatment at day 84. Mouse serum was monitored weekly for serum HBV DNA and HBV antigens.

Intracellular Metabolites of TAF

Primary human hepatocytes were purchased from Life Technologies (Grand Island, NY) and BioreclamationIVT (Baltimore, MD) were plated in collagen-coated 12-well tissue culture plates with Matrigel overlay seeded at confluency. The cells were cultured with Cell Maintenance Medium (Williams E supplemented with Primary Hepatocyte Maintenance Supplements) (Life Technologies) containing 1% DMSO and 2% Fetal Bovine Serum (FBS) in a 37 °C incubator under a humid atmosphere of 95% air/5%

CO_2 (v/v) for 3 or 4 days. The triplicate wells were incubated with 0.5 μM GS-7340 in the absence or presence of 1.5 μM of GS-SBA-1 dissolved in Cell Maintenance Medium. The inhibitor concentration of 1.5 μM is anticipated to be significantly greater than the projected clinical Cmax (bound + unbound) of GS-SBA-1. After a 24-hour continuous incubation, the untreated cells in several wells were trypsinized and counted to determine the cell numbers. The remaining cells were washed twice with 2.0 mL ice cold saline (0.9% sodium chloride) solution and then scraped into 0.5 mL 70% methanol containing 100 nM 2-chloro-adenosine-5'-triphosphate (Sigma-Aldrich, St. Louis, MO) as an internal standard. Samples were stored overnight at −20 °C to facilitate nucleotide extraction, centrifuged at 15,000 × g for 15 minutes and then supernatant was transferred to clean tubes for drying in a MiVac Duo concentrator (Genevac, Gardiner, NY). Dried samples were then reconstituted in 1 mM ammonium phosphate buffer (pH = 7) for analysis by LC-MS/MS as previously described (12, 13).

Determination of TFV, TFV-MP, and TFV-DP in Primary Human Hepatocytes

Analytes were separated using a 50 x 2 mm x 2.5 μm Luna C18(2) HST column (Phenomenex, Torrance, CA) connected to a LC-20ADXR (Shimadzu, Columbia, MD) ternary pump system and HTS PAL autosampler (LEAP Technologies, Carrboro, NC). A multi-stage linear gradient from 10% to 50% acetonitrile in a mobile phase containing 3 mM ammonium formate (pH 5.0) with 10 mM dimethylhexylamine at a flow rate of 150 μL/min was used to separate analytes. Detection was performed on an API 5000 (Applied Biosystems, Foster City, CA) MS/MS operating in positive ion and multiple reaction monitoring modes. TFV and TFV-DP was quantified using a 7-point standard curve ranging in concentration from 0.21 to 150 pmol/million cells prepared in cell extract from untreated primary human hepatocytes. TFV-MP was calculated using TFV-DP calibration curve.

HPLC Conditions

Time (min)	Flow Rate (mL/min)	Mobile Phase A (%)	Mobile Phase B (%)

1.00	0.15	80	20
3.00	0.15	72	28
6.00	0.15	0	100
6.50	0.15	0	100
6.51	0.15	80	20

Mass Spectrometry

Mass spectrometer: API 5000 triple quadrupole mass spectrometer (Applied Biosystems)

Operation mode: multiple reaction monitoring (MRM)

Mass spectrometry parameters

**Ion Source**	**Probe Height (mm)**	**Spray Voltage (V)**	**Temperature (°C)**	**Curtain Gas (psi)**	**Collision Gas (psi)**
ESI +	**5**	**5500**	**550**	**20**	**9**

MRM Channels

**Analyte**	**Description**	**Parent Mass (m/z)**	**Product Mass (m/z)**	**Declustering Potential (V)**	**Collision Energy (eV)**
GS-1278	Mono-phosphate	288.0	176.1	110	36
GS-77389	Di-phosphate	448.1	176.1	210	60
Chloro-ATP	Internal standard	541.9	169.9	291	39

Assay Performance

Calibration curve parameters

**Analyte**	**Sample Matrix**	**RT (min)**	**Range (pmol/million cells)**	**Weighting Index**	**R2**

GS-1278	Primary Human Hepatocytes	5.1	0.21 – 150	1/x	1.0
GS-77389		5.6	0.21 – 150	1/x	1.0

Synergy Studies

Three days after infection of PHH with AD38 virus (Day 0), maintenance medium was replenished and cells were dosed with compound supplied in 100% DMSO using the HP Digital Dispenser D300 (Hewlett Packard, Palo Alto, CA). For combination studies, GS-SBA-1 was serially diluted in eight steps of 1:2 dilutions in the horizontal direction with TAF serially diluted in five steps of 1:3 dilutions in the vertical direction. The EC_{50} value of each individual compound was selected as the midpoint for the concentration range tested. All combinations were performed in triplicate plates. Vehicle and compound containing wells were normalized such that the final concentration of DMSO was 1.7% (v/v). Medium containing compound was removed and PHH were re-dosed with new media containing the appropriate compounds on Day 3 following the initial dose for a total of 2 doses. On Day 6 post-initiation of dosing, amounts of extracellular viral DNA (vDNA) were measured to assess antiviral activity. Antiviral activity of each test compound was determined from the vDNA data by comparing compound-treated PHH to DMSO-treated PHH to generate a percent of DMSO control value (% DMSO control). The % DMSO control was calculated by the following equation: % DMSO Control = 100 × (Xc / XD) where Xc is the signal from the compound-treated cells and XD is the signal from the DMSO-treated cells. The % DMSO control for vDNA was plotted versus the log of each compound concentration in GraphPad Prism (version 6; GraphPad Software, La Jolla, CA) to generate dose-response curves. EC_{50} values were defined as the test compound concentration that caused a 50% decrease in vDNA. Dose-response curves were fitted using the nonlinear regression equation “log(agonist) versus response – Variable slope (four parameters)” in GraphPad Prism to determine EC_{50} values. The combination study experimental data were analyzed using the MacSynergy II software program developed by Prichard and Shipman (14).

References

1. Schuck P. 2000. Size-distribution analysis of macromolecules by sedimentation velocity ultracentrifugation and lamm equation modeling. Biophys J 78:1606-19.
2. Laue TM, Shah B, Ridgeway TM, Pelletier SL. 1992. Computer-aided Interpretation of Sedimentation Data for Proteins, Analytical ultracentrifugation in biochemistry and polymer science. Royal Society of Chemistry, Cambridge, U.K. pp. 90-125.
3. Layton CJ, Hellinga HW. 2010. Thermodynamic analysis of ligand-induced changes in protein thermal unfolding applied to high-throughput determination of ligand affinities with extrinsic fluorescent dyes. Biochemistry 49:10831-41.
4. Ladner SK, Otto MJ, Barker CS, Zaifert K, Wang GH, Guo JT, Seeger C, King RW. 1997. Inducible expression of human hepatitis B virus (HBV) in stably transfected hepatoblastoma cells: a novel system for screening potential inhibitors of HBV replication. Antimicrob Agents Chemother 41:1715-20.
5. Niu C, Livingston CM, Li L, Beran RK, Daffis S, Ramakrishnan D, Burdette D, Peiser L, Salas E, Ramos H, Yu M, Cheng G, Strubin M, Delaney WI, Fletcher SP. 2017. The Smc5/6 Complex Restricts HBV when Localized to ND10 without Inducing an Innate Immune Response and Is Counteracted by the HBV X Protein Shortly after Infection. PLoS One 12:e0169648.
6. Livak KJ, Schmittgen TD. 2001. Analysis of relative gene expression data using real-time quantitative PCR and the 2(-Delta Delta C(T)) Method. Methods 25:402-8.
7. Cai D, Nie H, Yan R, Guo JT, Block TM, Guo H. 2013. A southern blot assay for detection of hepatitis B virus covalently closed circular DNA from cell cultures. Methods Mol Biol 1030:151-61.
8. Dobin A, Davis CA, Schlesinger F, Drenkow J, Zaleski C, Jha S, Batut P, Chaisson M, Gingeras TR. 2013. STAR: ultrafast universal RNA-seq aligner. Bioinformatics 29:15-21.
9. Yang H, Qi X, Sabogal A, Miller M, Xiong S, Delaney WEt. 2005. Cross-resistance testing of next-generation nucleoside and nucleotide analogues against lamivudine-resistant HBV. Antivir Ther 10:625-33.
10. Qi X, Xiong S, Yang H, Miller M, Delaney WEt. 2007. In vitro susceptibility of adefovir-associated hepatitis B virus polymerase mutations to other antiviral agents. Antivir Ther 12:355-62.
11. Tateno C, Yoshizane Y, Saito N, Kataoka M, Utoh R, Yamasaki C, Tachibana A, Soeno Y, Asahina K, Hino H, Asahara T, Yokoi T, Furukawa T, Yoshizato K. 2004. Near completely humanized liver in mice shows human-type metabolic responses to drugs. Am J Pathol 165:901-12.
12. Murakami E, Wang T, Park Y, Hao J, Lepist EI, Babusis D, Ray AS. 2015. Implications of efficient hepatic delivery by tenofovir alafenamide (GS-7340) for hepatitis B virus therapy. Antimicrob Agents Chemother 59:3563-9.
13. Delaney WEt, Ray AS, Yang H, Qi X, Xiong S, Zhu Y, Miller MD. 2006. Intracellular metabolism and in vitro activity of tenofovir against hepatitis B virus. Antimicrob Agents Chemother 50:2471-7.
14. Prichard MN, Aseltine KR, Shipman CJ. 1993. MacSynergy II. Version 1.0. User's manual. University of Michigan, Ann Arbor.

Supplemental Table 1. Antiviral activity of GS-SBA-1 in HBV-infected PHH treated in therapeutic format

		Mean Extracellular HBV DNA EC_{50}, µM		Cytotoxicity CC_{50}, µM	
		**GS-SBA-1**	**TFV**	**GS-SBA-1**	**TFV**
PHH[a]	Donor A	0.015 ± 0.012	0.026 ± 0.0048	>2	>2
	Donor B	0.018	0.025	>2	>2
	Donor C	0.019	0.035	>2	>2
	Donor D	0.021	0.032	>2	>2
	All Donors	0.019 ± 0.0071	0.030 ± 0.0077	>2	>2
HepG2-NTCP[b]		0.0093 ± 0.0035	0.00075*	>2	>2

The assay schematic is depicted in Figure 2A.
[a]EC_{50} calculated by 4-parameter logistic curve fitting data from 6-day treatment of HBV-infected PHH. The top of the curve was constrained to 100. Averages ± SD are shown for four independent experiments for donor A and averages of two independent experiments for donors B through D are shown.
[b]6-day treatment of HBV-infected HepG2-NTCP cells. The top of the curve was constrained to 100. Averages ± SD are shown across four independent experiments for GS-SBA-1 and average of two independent experiments for TFV are shown.
*HepG2-NTCP cells were treated with TAF

Supplemental Table 2. Antiviral activity of GS-SBA-1 in PHH treated at the time of HBV infection

Assay, EC_{50}/CC_{50} μM[a]	GS-SBA-1				TFV			
	PHH Donor				PHH Donor			
	A	B	C	D	A	B	C	D
**HBV DNA**	0.0072	0.0062	0.011	0.0057	0.012	0.020	0.022	0.012
**HBV RNA**	0.12	0.13	0.12	0.097	>2			
**HBsAg**	0.12	0.20	0.080	0.14	>2			
**HBeAg**	0.19	0.20	0.12	0.16	>2			
**Cytotoxicity**	>2				>2			

The assay schematic is depicted in Figure 3A.
[a]Calculated by 4-parameter logistic curve fitting of data from 6-day treatment of HBV-infected PHH. The top was constrained to 100%. Averages of two independent experiments are shown.

Supplemental Table 3. Primers sequences used in the study

**Name**	**Sequence (5’-3’)**
HBx forward primer	GGACCCCTGCTCGTGTTACA
HBx reverse primer	GAGAGAAGTCCACCACGAGTCTAGA
HBx probe	[6FAM] TGTTGACAAGAATCCTCACCAATACCAC [NFQ-MGB]
HBV-CCCF	CCGTGTGCACTTCGCTTCA
HBV-CCCR	GCACAGCTTGGAGGCTTGA
HBV-CCC probe	[6FAM] CATGGAGACCACCGTGAACGCCC

Supplemental Figure 1

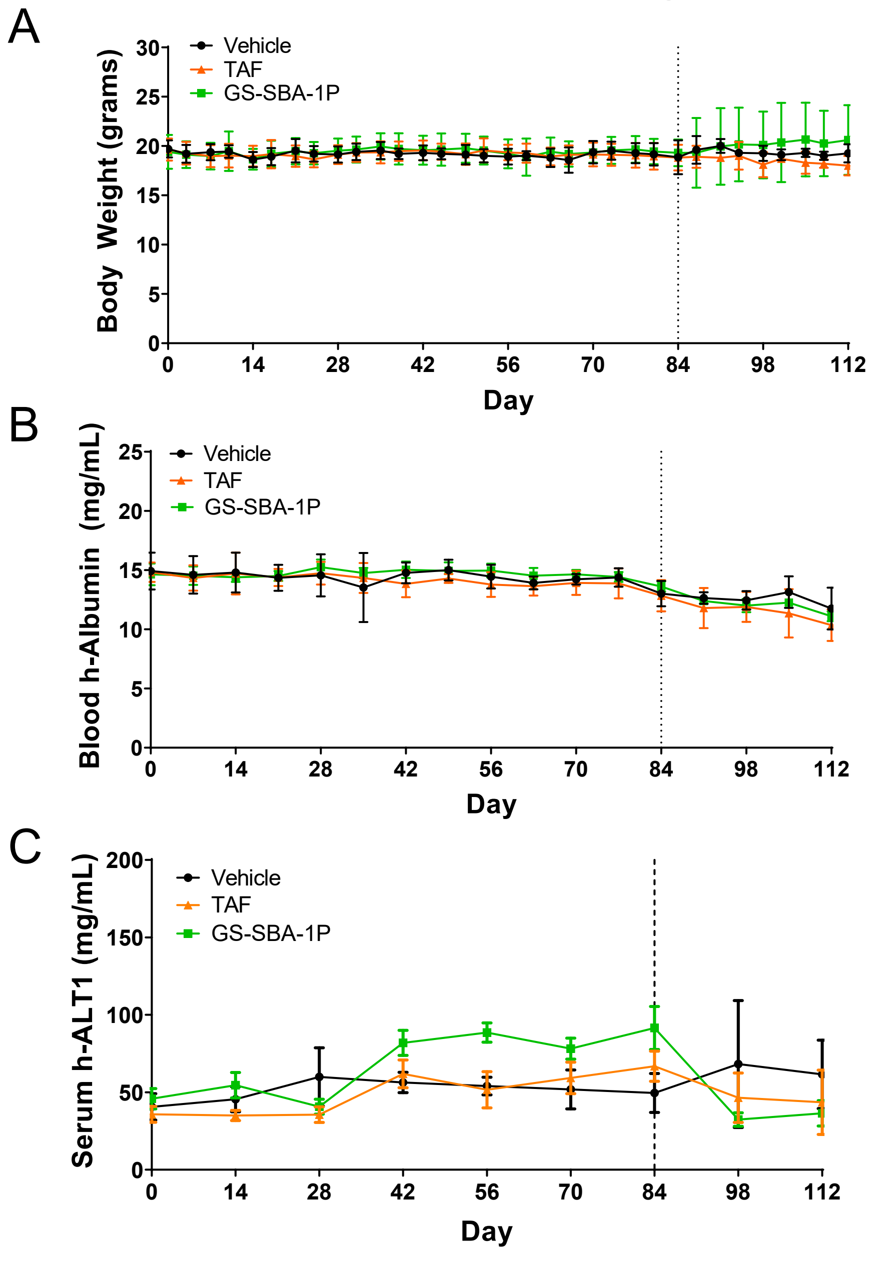

Supplemental Figure 1. Antiviral efficacy of GS-SBA-1 in HBV-infected uPA/SCID mice. Fifteen uPA/SCID mice were infected with genotype C HBV. After 8 weeks of infection, mice were dosed with 100 mg-eq/kg GS-SBA-1P or 5 mg-eq/kg TAF PO once daily (n=5 per group) for 84 days. Mice were followed for an additional 28 days. Longitudinal analysis of (A) body weight, (B) blood albumin and (C) serum human ALT1 levels during treatment and after treatment follow-up. All data are presented as mean ± SD.
